# How the COVID-19 pandemic has affected eating habits and physical activity in breast cancer survivors: the DianaWeb study

**DOI:** 10.1007/s11764-022-01294-w

**Published:** 2022-12-13

**Authors:** Mattia Acito, Tommaso Rondini, Giuliana Gargano, Massimo Moretti, Milena Villarini, Anna Villarini

**Affiliations:** 1 Department of Pharmaceutical Sciences, University of Perugia, Via del Giochetto, 06122 Perugia, Italy; 2 Department of Research, Fondazione IRCCS Istituto Nazionale Dei Tumori, Via Giacomo Venezian 1, 20133 Milan, Italy; 3 Department of Medicine and Surgery, University of Perugia, Piazzale Settimio Gambuli, 06132 Perugia, Italy

**Keywords:** Breast cancer, COVID-19 pandemic, DianaWeb, Mediterranean diet, Physical activity

## Abstract

**Purpose:**

Breast cancer (BC) survivors are increasingly interested in learning about healthy lifestyles to reduce the risk of BC recurrence and mortality. The DianaWeb study, a community-based participatory research, offers BC patients a specific interactive website to help them in adopting and maintain correct lifestyles, in line with World Cancer Research Fund recommendations. However, to limit the spread of the COVID-19 pandemic, most countries introduced restrictions which, inevitably, caused sudden lifestyle changes.

The current study aimed at evaluating changes in lifestyle in BC survivors before, during, and after the first two waves of the COVID-19 pandemic.

**Methods:**

We used data of 224 BC cancer survivors enrolled in the DianaWeb study. We evaluated the adherence to physical activity (PA) guidelines, to Mediterranean diet (MD), and WCRF recommendations, at baseline, during and after the first two waves of the COVID-19 pandemic. We estimated the association between PA, MD, and WCRF adherence with sociodemographic characteristics, years from enrolment in the DianaWeb study, and type of breast cancer.

**Results:**

As expected, during confinement periods, we observed a significant decrease in walking activity and in the percentage of patients performing vigorous PA. In post-quarantine, total weekly energy expenditure increased significantly. BC patients participating in the DianaWeb study for more than 3 years were more likely to be more active. There were no changes in MD and WCRF adherence.

**Conclusions:**

Our results show that the proposed interactive website was useful in achieving durable lifestyle changes, that have not been undermined either during the COVID-19 pandemic.

**Implications for Cancer Survivors:**

Patient education is essential for guiding BC survivors toward improving their health outcomes; therefore, an interactive website like the one proposed by the DianaWeb study may be useful to improve healthy lifestyles.

**Supplementary Information:**

The online version contains supplementary material available at 10.1007/s11764-022-01294-w.

## Introduction

Breast cancer (BC) is the most common cancer worldwide, and the leading cause of cancer death among women. Recent data by the GLOBOCAN project show that new cases of BC in 2020—worldwide and at all ages—were about 2.3 million, with an incidence of 11.7% of all types of cancer [1]. BC mortality is estimated to be about 6.9%, with 684,996 deaths worldwide in 2020 [2]. BC survival for at least 5 years after diagnosis varies between 90% or more in high-income countries and 66% in India and 40% in South Africa. BC is the world’s most prevalent cancer: at the end of 2020 there were 7.8 million women alive who were diagnosed with BC in the past 5 years [3]. BC is the most widespread and frequent cancer in Italy, with new diagnoses amounting to more than 55,000 annually and an estimated 12,500 deaths. In total, 834,000 women lived with a breast cancer diagnosis in Italy in 2020 [4]. In Italy, because of the availability of freely accessible screening programs and awareness among women and general practitioners, most breast malignancies are diagnosed at an early stage of the disease. This means that the 5-year survival rate is very high (87%, one of the highest recorded in Europe) [5].

Understanding biochemical and metabolic parameters playing active roles in BC prevention and recurrence is a key point in the management of this pathology in a broad sense. Particularly, this was the aim of several clinical trials carried out in the last 25 years, named under the acronym of DIANA (DIet ANd Androgens) intervention studies. These studies found out that metabolic syndrome and high serum insulin and testosterone levels—known risk factors for BC incidence and possibly for BC recurrence as well [6, 7]—might be taken under control by following a Mediterranean-inspired diet and practicing regular physical activity (PA) [8, 9].

Indeed, all the abovementioned risk factors are clearly—or at least, partially—related to an unhealthy lifestyle and can be favorably modified by adhering to the European Code Against Cancer (ECAC) and the World Cancer Research Fund/American Institute for Cancer Research (WCRF/AICR) recommendations. To lower the risk of BC recurrences and mortality, the WCRF [10, 11], the ECAC [12], and the American Cancer Society (ACS) [13] recommend following a dietary pattern high in vegetables, fruits, and whole grains, as well as an exercise regimen including 150 min per week of moderate-intensity exercise or 75 min of vigorous-intensity exercise plus two strength training sessions per week. Some BC survivors may have difficulty meeting these recommendations, and data suggest that only 18 and 37% of BC survivors meet nutrition and PA guidelines, respectively [14]. Despite an overall improvement in survival rates, mostly due to technological and clinical advances in early diagnosis and treatment [15], BC has a dramatic impact on patients’ lives, accounting for 17.7 million disability-adjusted life years (DALYs) worldwide in 2017 [16].

In this context, in response to an increase in BC survivors’ prevalence, in the last years, researchers and clinicians also focused on improving cancer survivors’ quality of life. Maintaining high PA levels is known to play a role in extending the healthy lifespan of BC survivors and in improving their health-related quality of life [17–19]. Mediterranean diet (MD), to which is attributed an anti-inflammatory potential, has been proposed to possess a potential protective effect for BC mortality [20, 21]. Although PA and MD are inexpensive and largely beneficial preventive measures, information about salutogenic lifestyles is not currently available to patients and is not yet included in oncology protocols in several countries, including Italy [22]. With a few exceptions [23], physicians are not aware of these scientific results and are not yet culturally prepared for lifestyle prescriptions.

This knowledge gap cannot be further accepted, and for this reason, the DIANA trials were implemented through the DianaWeb study, a community-based participatory research (CBPR) that offers BC patients a specific interactive website where patients themselves can find evidence-based recommendations and tips for sustaining lifestyle changes [24]. The DianaWeb study also aims at monitoring lifestyle changes over time, to verify whether a high adherence to MD and WCRF recommendations can significantly reduce important risk factors—such as factors characterizing the metabolic syndrome—for BC recurrences/metastasis and ultimately increase survival in women with BC diagnosis.

However, since March 2020, the outbreak of COVID-19 (Coronavirus disease 2019), caused by a new type of coronavirus (i.e., SARS-CoV-2) has deeply influenced—and is still influencing—our society by profoundly modifying in few days the usual behavior of families and individuals. Indeed, to limit the spread of the COVID-19 pandemic, most countries introduced restrictions. In Italy, the first lockdown period started on March 9, 2020, and lasted until May. Due to epidemiological context, a second lockdown was also imposed in November 2020 and lasted until April 2021. Lockdown rules limited the mobility of citizens to the necessary activities in everyday life (commuting to work, and shopping for basic needs), stopped most nonessential services, significantly reduced the number of people allowed to stay in a single room (e.g., in shops or public transport), and imposed an obligation to wear filtering facepieces to cover mouth and nose in public spaces. Such measures translated into self-isolation and social distancing, deeply affected, among others, the possibility of doing regular PA, and potentially reduced access to healthy food [25]. Given the beneficial role of lifestyle in BC prevention and recurrence, the pandemic phenomenon should also be studied for its indirect consequences in a such vulnerable population.

The objective of the present study was to evaluate changes in PA levels and eating habits in the DianaWeb cohort during and after the period of social isolation due to the first two waves of the COVID-19 pandemic, with respect to the pre-pandemic period. Furthermore, we analyzed the potential variables that may have influenced the lifestyle modifications.

## Methods

### Study design

The DianaWeb study is a multicentric CBPR that uses a specific interactive website (www.dianaweb.org) developed by It’s Informatica e Comunicazione Srl (Brescia, Italy). The study is offered to BC patients, whatever the clinical stage of their disease, and is designed to understand needs, fill knowledge gaps, and help women with BC to maintain WCRF/AICR, ECAC, and ACS-based lifestyles.

Detailed information regarding the study protocol has been reported elsewhere [24].

Briefly, patients are recruited on a voluntary basis, and, after having signed an informed consent form, they are enrolled in the study. Once registered, patients are requested to complete—twice a year— online questionnaires to gather information about: (a) demographic and anthropometric data (body weight, body height, and waist circumference); (b) medical history; (c) MD adherence; (d) PA levels; (e) results of routine biochemical analysis (during pandemic, very few observations could be collected, and thus these data were not considered in the analysis performed for this study).

Through the website, participants are informed about the most recent scientific evidence regarding BC recurrences prevention and are encouraged to adhere to the lifestyle recommendations proposed by the WCRF. On the website, these recommendations are integrated, among others, with information on characteristics of nutrients, recipes, and calls to perform PA of moderate intensity. As social and convivial aspects are crucial in this project, kitchen classes, walking groups, and meetings concerning the latest evidence-based recommendations for a healthy lifestyle are organized by the staff, who actively participates in these activities.

The social distancing policy is one of the recommended policies by the WHO to stop the transmission of SARS-CoV-2, and for this reason, all the activities of the DianaWeb study were shifted from in-person to online.

Because the emotional responses to the COVID-19 pandemic had notable impacts on food-related attitudes, PA, and behaviors, to prevent potential deviations from WCRF recommendations, during the lockdown periods, the remote cooking shows, conferences, and remote PA promotion of the DianaWeb study were implemented and offered every week.

## Participants

The DianaWeb is an open cohort established in September 2016, and, at present (November 2022), a total of 1,711 patients are enrolled in the study. For the present study, the population was composed of 224 women (DianaWeb pandemic sub-cohort) that completed the questionnaires immediately before, during, and after the COVID-19 pandemic waves.

## Data collection

### Basic information

Data were collected (a) at baseline; (b) 2 months before the COVID-19 pandemic (January and February 2020); (c) during the Italian lockdowns (March–May 2020 and November 2020–April 2021); (d) and during post-lockdown periods with easing of restrictions (June–October 2020 and May–July 2021).

Basic information including residence, smoking habits, education level, marital status, medical history, and results of routine blood tests were collected. The patients were classified for residence as living in Northern, Central, or Southern Italy. The education variable was trichotomized into “first-level” (lasting 8 years, for children from 6 to 14 years of age), “second level” (lasting 5 years for students from 14 to 19 years of age), and higher education (offered by universities, institutes of the Higher Education in Art and Music system, and Higher Technical Institutes).

### Physical activity

The International Physical Activity Questionnaire—Short Form (IPAQ-SF) was used to assess PA level. The IPAQ-SF (9 generic items) records the activity of four intensity levels: vigorous-intensity activity, moderate-intensity activity, walking, and sitting. The IPAQ-SF sum score was expressed in PA Metabolic Equivalent of Task (MET)-minutes per day or week. Total weekly PA MET-minutes were estimated by adding up the calculated MET-minutes within each PA intensity level (moderate intensity = 4.0 MET, vigorous intensity = 8.0 MET, and walking = 3.3 MET). MET-min/week was used as a general indicator of low active (MET < 600), moderately active (MET ≥ 600), or high active (MET ≥ 3000) patients [26].

### MD adherence

A 17-item MedDiet questionnaire—used in the PREDIMED-PLUS trial—was employed to assess adherence to MD. Compliance with each of the 17 items of the questionnaire was scored with 1 point; otherwise, the score was 0 points. The MedDiet score ranged from 0 to 17, and adherence to MD was categorized into 4 categories: low (total score ≤ 6), low to moderate (total score between 7 and 8), moderate to high (total score between 9 and 10), and high (total score between 11 and 17) [27].

### 24-h dietary recall

To better evaluate the patients’ compliance with the WCRF dietary recommendations, the enrolled women were asked to fill in a 24-h dietary recall containing a list of 42 food items organized into 6 groups: drinks; milk, and dairy products; sweets and confectionery; bread and grains; meat, fish, eggs, and meat substitutes; legumes, vegetables, fresh and dried fruit, nuts, and seeds; sauces, animal, and vegetable fats. The women had to indicate only whether, on the previous day, had eaten or not the specified food at breakfast, lunch, dinner, and breaks, without any information on portion size or weight. As suggested by Bruno et al. [28], we calculated two variables, recommended food consumption/day (RFC) and discouraged food consumption/day (DFC), by putting together all recommended and all discouraged foods eaten in the past 24 h, respectively. White meat, eggs, coffee, unsweetened citrus juices, and unsweetened fruit juices were considered neutral food (not recommended but not discouraged). We analyzed the magnitude of changes in RFC and DFC consumption by evaluating, for each woman, the difference (delta, Δ) between servings declared during the three pandemic periods (before, during, and after) and at baseline.

### Adherence to WCRF indications

Finally, adherence to the WCRF indications was estimated by operationalizing 6 of the 8 items proposed by Shams-White et al. [29]. The 6 items evaluated in this work are linked to the following domains: (a) be a healthy weight, (b) be physically active, (c) eat a diet rich in whole grains, vegetables, fruit, and beans, (d) limit the intake of red meat and avoid processed meat, (e) limit consumption of sugar-sweetened drinks, (f) limit alcoholic drinks and the consumption of salt and salt-preserved foods. For each of the 6 items, a maximum score of 1 was assigned when the recommendations were fully satisfied, a value of 0 when the recommendations were not satisfied, and 0.5 points as an intermediate score. We were not able to evaluate breastfeeding and consumption of ultra-processed foods because this information was not available in our study.

## Statistical analysis

Categorical variables were summarized as frequency and percentage, whereas mean and standard deviation (SD) were calculated for continuous variables.

The Cochran’s Q test was used to compare qualitative data, whereas ANOVA was used to compare means of normally distributed quantitative data. In the case of statistically significant *F* statistics, ANOVA was followed by a Dunnet post hoc analysis. Pearson’s correlation coefficient was calculated to assess the strength and direction of the linear relationships between pairs of variables normally distributed. For non-ordinal variables, Spearman’s correlation coefficient was calculated.

ß-Coefficients with 95% confidence intervals (95% CI) from generalized linear model (GLM) were used to estimate the association between changes in PA, MD, and WCRF adherence with sociodemographic characteristics, years from enrolment in the DianaWeb study, and type of breast cancer (triple-negative).

All statistical analyses were carried out with SPSS software for Windows (version 20.0; SPSS Inc., Chicago, IL, USA), and *p* values < 0.05 were considered statistically significant.

## Results

Table 1 reports some descriptive characteristics of the women enrolled in the DianaWeb study (minus the DianaWeb pandemic sub-cohort) until July 2021 and women included in the DianaWeb pandemic sub-cohort. We found that the 2 groups were very similar in most of the demographic characteristics. In both groups, the mean age of participants was about 55 years, and most of the participants (about 70%) were aged 41 to 60. The majority of patients were married and living in Northern Italy. About half of the women reported having a university degree. The patients were followed up, on average, for about 3 years, whereas only 255 patients (17.1%) in the DianaWeb study and 42 patients (18.8%) in the DianaWeb pandemic sub-cohort were followed up for more than 5 years.

**Table 1:** Descriptive characteristics of the full DianaWeb cohort and the DianaWeb pandemic sub-cohort

Characteristics	Full DianaWeb cohort ^a^ (*n* = 1,487)	DianaWeb pandemic sub-cohort (*n* = 224)	*p*
Age ^b^	55.06 ± 8.81	55.49 ± 8.63	0.491 ^d^
Young adults (aged 21–40) ^c^	66 (4.5)	9 (4.0)	0.862 ^e^
Adults (aged 41–60) ^c^	1.040 (69.9)	156 (69.6)	0.938 ^e^
Over 60 aged ^c^	381 (25.6)	59 (26.3)	0.806 ^d^
Marital status ^c^			
Never married	325 (21.8)	55 (24.6)	0.387 ^e^
Widowed	41 (2.8)	6 (2.7)	1.000 ^e^
Separated/divorced	180 (12.1)	16 (7.1)	0.031 ^e^
Married	941 (63.3)	147 (65.6)	0.550 ^e^
Education level ^c^			
First level	78 (5.3)	14 (6.2)	0.525 ^e^
Second level	697 (46.9)	91 (40.6)	0.083 ^e^
Higher education	711 (47.8)	119 (53.1)	0.150 ^e^
Area of residence			
Northern Italy	1,093 (73.5)	173 (77.2)	0.253 ^d^
Central Italy	281 (18.9)	30 (13.4)	0.051 ^e^
Southern Italy	113 (7.6)	21 (9.4)	0.351 ^e^
Years from enrolment in DianaWeb study ^b^	3.00 ± 1.58	3.13 ± 1.33	0.230 ^d^
≤ 2 years ^c^	580 (39.0)	92 (41.1)	0.558 ^e^
3–4 years ^c^	652 (43.8)	90 (40.2)	0.312 ^e^
≥ 5 years ^c^	255 (17.1)	42 (18.8)	0.570 ^e^

^a^Subjects of the DianaWeb pandemic sub-cohort not included

^b^Results expressed as the mean ± SD

^c^Results expressed as the number of subjects, the percentage between brackets

^d^Student’s *t* test: full DianaWeb cohort vs DianaWeb pandemic sub-cohort

^e^χ^2^ test: full DianaWeb cohort vs DianaWeb pandemic sub-cohort

Most of the women in the DianaWeb cohort and the pandemic sub-cohort have been diagnosed with BC for 5 years or more, almost one-third of the whole cohort for more than 10 years. Overall, in the DianaWeb study cohort, the percentage of left-sided breast cancer patients was 50.9, not significantly different from the pandemic sub-cohort (49.6%). Bilateral synchronous breast cancer accounted for about 3% of all breast cancers in both groups. In the DianaWeb cohort and the pandemic sub-cohort, the most common tumor stage was IA, and the most common tumor grade was G2. A large proportion of patients in both groups was ER and PR positive, whereas 506 patients in the DianaWeb cohort (34.0%) and 90 patients in the DianaWeb sub-cohort (40.2%) were HER2 positive. Triple-negative phenotype accounted for 23.7% of the whole DianaWeb cohort and 28.1 of the pandemic sub-cohort, a proportion higher than that reported for the Italian population (15–20%) (Supplementary Table 1) [30].

The PA level at baseline and along the three periods examined in the DianaWeb sub-cohort is reported in Tab. 2. As regards vigorous PA, we found an increase in the number of physically active women immediately before the COVID-19 pandemic (29.5%) when compared with baseline (8.5%). During the lockdown, we observed a statistically significant decrease in the percentage of subjects who declared to perform vigorous PA, compared with data observed before the COVID-19 pandemic. At the same time, the average MET-min/week increased significantly during lockdown (from 1099.27 MET-min/week before the COVID-19 pandemic to 1543.37 MET-min/week). This trend was also observed for moderate PA, with a significant increase in MET-min/week during lockdown compared with the period before. The level of MET-min/week related to walking activity during lockdown—along with a significant decrease in the percentage of people who performed it—was probably related to more frequent and intense use of indoor equipment, such as a treadmill or similar, by a limited group of women. Probably, subjects who decided to practice PA at home tended to perform vigorous exercise, as suggested by the weekly exercise webinars we offered to this cohort.

**Table 2:** Level (expressed as MET-min/week) and percentage of declared specific physical activity at baseline and immediately before, during, and after lockdown periods, in the DianaWeb pandemic sub-cohort

	Baseline	Before COVID-19 pandemic	During lockdown periods	After lockdown periods
Vigorous PA (total) ^a^				
Declared ^b^	19 (8.5)	66 (29.5) ^☨^	38 (17.0) ^†^	74 (33.0) ^☨^
Vigorous PA (within declaring group)	1795.37 ± 964.06	1099.27 ± 937.90 ^§^	1543.37 ± 1020.06 *	1356.65 ± 943.02
Moderate PA (total) ^a^				
Declared ^b^	91 (40.6)	109 (48.7)	103 (46.0)	133 (59.4) ^☨^
Moderate PA (within declaring group)	1086.73 ± 925.25	1092.48 ± 958.98	1407.73 ± 1021.58*	1163.46 ± 914.10
Walking (total) ^a^				
Declared ^b^	190 (84.80)	192 (85.7)	137 (61.2) ^†☨^	197 (87.9)
Walking (within declaring group)	761.51 ± 675.79	876.82 ± 851.92	769.60 ± 803.85	906.16 ± 870.25
Total PA ^a^				
Declared ^b^	206 (92.0)	203 (90.6)	177 (79.0) ^☨†^	224 (100.0)
Total PA (within declaring group)	1348.02 ± 1264.13	1773.31 ± 1404.30 ^§^	1746.21 ± 1419.90 ^§^	1935.92 ± 1616.27 ^§^
< 600 MET ^b^	94 (42.0)	68 (30.4) ^☨^	89 (39.7) ^†^	49 (21.9) ^☨^
600–3000 MET ^b^	106 (47.3)	116 (51.8)	108 (48.2)	129 (57.6) ^☨†^
≥ 3000 MET ^b^	24 (10.7)	40 (17.9)	27 (12.1)	46 (20.5) ^☨^

^a^Results expressed as the mean ± SD

^b^Results expressed as the number of subjects, the percentage between brackets

^§^ANOVA, *p* < 0.05 before, during, or after lockdown vs baseline

^*^ANOVA, *p* < 0.05 during or after lockdown vs before COVID-19 pandemic

^☨^Cochran’s Q test, *p* < 0.05 before, during, or after lockdown vs baseline

^†^Cochran’s Q test, *p* < 0.05 during or after lockdown vs before COVID-19 pandemic

Overall, the total PA shifted from 1348.02 ± 1264.13 MET-min/week at baseline to 1773.31 ± 1404.30 MET-min/week immediately before the COVID-19 pandemic (*p* > 0.05). During lockdown periods, we did not observe any significant decrease in total MET-min/week. Indeed, during lockdown periods, sedentary women (< 600 MET-min/week) significantly increased. The post-lockdown relaxation of restrictions determined a statistically significant decrease in inactive subjects and a statistically significant increase in highly active women, compared with baseline.

Even if some studies suggest that there is a high prevalence of sleep problems during the COVID-19 pandemic [31], in our cohort the sleep quality was not compromised by the pandemic, and any statistically significant difference with baseline values was observed. The evaluation of smoking status showed that in the DianaWeb pandemic sub-cohort 4.9% of women used to smoke. In the full DianaWeb cohort, the smokers were 7.1%, not significantly different when compared with the pandemic sub-cohort, *p* = 0.252. When compared with Italian women, the percentage of smokers in the DianaWeb cohort was lower (15.3 vs 7.1%, respectively) [32]; however, these data were expected as most BC patients quit or attempt to quit shortly after a cancer diagnosis [33]. The number of cigarettes smoked every day was not significantly influenced by the pandemic period, even if we observed a minimal reduction during the lockdown (23 ± 16 cigarettes per day) (Supplementary Table 2).

International guidelines for a healthy diet recommend a high intake of whole grains, pulses, fruit, and vegetables and limit high-calorie foods with high percentages of simple sugars and fats, red meat, and high salt consumption [11, 12]. As all these recommendations are attributable to MD, we also investigated adherence to this diet pattern of subjects involved in this study.

The MedDiet score (Table 3) significantly increased consequently to DianaWeb nutritional intervention, and results showed that immediately before, during, or after lockdown periods, the great majority of the cohort had high adherence to MD (average values ranging from 60.7 to 66.5%). Less than 4.5% had a low adherence over the three pandemic periods. Interestingly, adherence to MD was not statistically affected by the COVID-19 pandemic.

**Table 3:** MD score and daily frequency consumption servings of recommended, discouraged, and non-recommended and not discouraged foods, at baseline and immediately before, during, and after lockdown periods, in the DianaWeb sub-cohort

	Baseline	Before COVID-19 pandemic	During lockdown periods	After lockdown periods
MedDiet ^a^	8.20 ± 1.88	10.06 ± 2.29 ^§^	9.75 ± 2.23^§^	9.92 ± 2.27^§^
≤ 5 ^b^	17 (7.6)	8 (3.6) ^☨^	10 (4.5) ^☨^	9 (4.0) ^☨^
6–9 ^b^	150 (67.0)	67 (29.9) ^☨^	78 (34.8) ^☨^	69 (30.8) ^☨^
≥ 10 ^b^	57 (25.4)	149 (66.5) ^☨^	136 (60.7) ^☨^	146 (65.2) ^☨^
Foods				
Recommended ^a^	10.73 ± 2.94	12.67 ± 5.50 ^§^	11.80 ± 3.48 ^§^	11.82 ± 3.46 ^§^
Discouraged ^a^	2.81 ± 2.15	2.69 ± 1.94	2.51 ± 1.73	2.44 ± 1.64
Not recommended and not discouraged ^a^	0.47 ± 0.29	0.50 ± 0.31	0.49 ± 0.24	0.50 ± 0.23

^a^Results expressed as the mean ± SD

^b^Results expressed as the number of subjects, the percentage between brackets

^§^ANOVA, *p* < 0.05 before, during, or after lockdown vs baseline

^*^ANOVA, *p* < 0.05 during or after lockdown vs before COVID-19 pandemic

^☨^Cochran’s Q test, *p* < 0.05 before, during, or after lockdown vs baseline

^†^Cochran’s Q test, *p* < 0.05 during or after lockdown vs before COVID-19 pandemic

Average recommended food servings per day ranged from 12.67 (before the pandemic) to 11.80 (during lockdown periods), whereas average daily discouraged food servings ranged from 2.69 (before the pandemic) to 2.44 (after lockdown periods). The frequency of servings of each food group did not change significantly along the three considered periods (*p* > 0.05), indicating a stable behavior in food choice. On the contrary, we observed a statistically significant increase in the consumption of recommended foods between the baseline and the three periods considered.

**Fig. 1:**
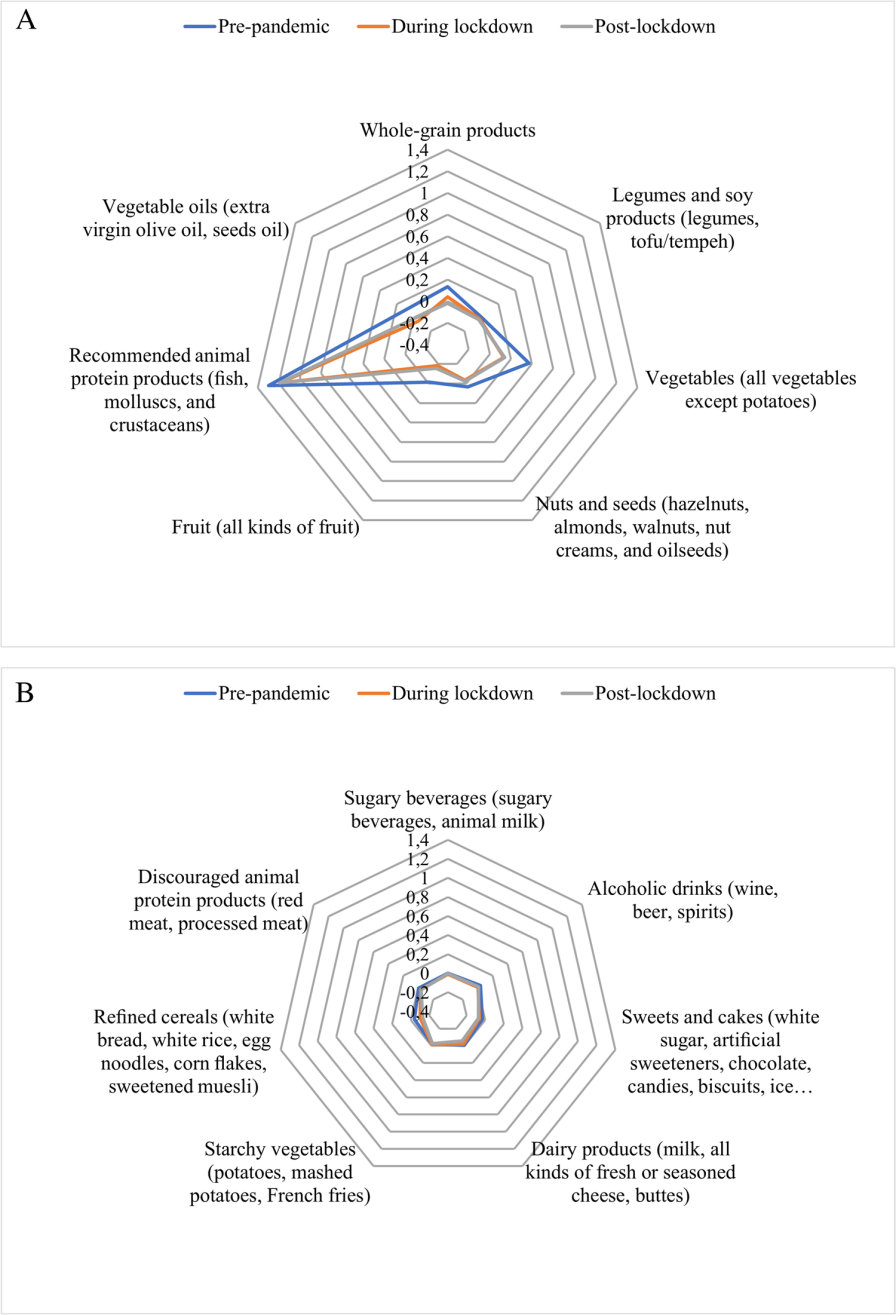
Changes in food frequencies consumption (**A**, recommended foods; **B**, discouraged foods) compared with baseline. The distance from the center of the point marked on the radius is the delta change of frequencies of food group consumption (servings/day)

Figure 1 shows changes in consumption of encouraged (Fig. 1/A) and discouraged (Fig. 1/B) foods (Δ food frequency was obtained from 24 h food frequency diaries), compared with baseline. Concerning baseline, immediately before the lockdown, we observed an increase in the number of daily servings consumed for all 7 encouraged food groups. The increase was statistically significant only for the consumption of fish, mollusks, and crustaceans.

During lockdown periods, the women of the DianaWeb cohort mildly decreased their consumption of vegetables, fruits, whole grain-product, legumes, and soy products. The consumption of the discouraged foods was reduced when compared with baseline and superimposable during the three pandemic periods.

WCRF score consisted of three components: (i) diet, (ii) body weight, and (iii) physical activity. The mean of the WCRF score showed a high adherence of women to recommendations, which is maintained constantly from the period before the pandemic’s beginning, to the end of the lockdown, indicating that women kept on pursuing a healthy lifestyle (Table 4).

**Table 4:** WCRF score at baseline and immediately before, during, and after lockdown periods, in the DianaWeb sub-cohort

	Baseline	Before COVID-19 pandemic	During lockdown periods	After lockdown periods
WCRF ^a^	4.15 ± 0.85	4.27 ± 0.78	4.31 ± 0.78	4.42 ± 0.76^§^
≤ 2 ^b^	12 (5.4)	9 (4.0)	10 (4.5)	6 (2.7)
3–4 ^b^	166 (74.1)	164 (73.2)	156 (69.6)	149 (66.5)
≥ 5 ^b^	46 (20.5)	51 (22.8)	58 (25.9)	69 (30.8)

^a^Results expressed as the mean ± SD

^b^Results expressed as the number of subjects, the percentage between brackets

^§^ANOVA, *p* < 0.05 before, during, or after lockdown vs baseline

^*^ANOVA, *p* < 0.05 during or after lockdown vs before COVID-19 pandemic

To determine factors associated with PA, and adherence to MD or WCRF recommendations, a GLM analysis was made (Supplementary Tables 3–5).

Overall, subjects included in the DianaWeb study from over the last 5 years resulted more physically active than those recruited for less than 2 years. Women who have been enrolled in the study for more than five years (Supplementary Table 3) present a statistically significant increased probability of 30% (before the pandemic), 40% (during lockdown), and almost 50% (after lockdown) of practicing correctly PA rather than women who were following the study from only 2 years or less. At baseline, when compared with women resident in Northern Italy, women resident in Southern Italy showed a 6% lower probability of adhering to the PA guidelines. After the inclusion in the DianaWeb study, a higher probability of adhering to the PA guidelines was observed in women living in Southern Italy (β coefficient before the pandemic = 1.140; 95% CI 1.126–1.153). Especially during the lockdown period, women who lived in Central or Southern Italy, rather than ones in Northern Italy—probably due to the possibility to get out, which was more restrictive through “red areas”—showed a higher inclination to practice PA. GLM analysis revealed that, at baseline, older age was associated with higher MET values. Based on the age classifications, the total PA significantly decreased during lockdown periods in over 60 women. As regards the educational level, data at baseline suggest that the odds of engaging in PA were higher for individuals with lesser education levels, compared with individuals with higher education. During the DianaWeb study, women with higher education get a greater chance of correctly practicing PA, even if during lockdown we could observe an increased inclination to PA in lower educational levels as well, possibly because of weekly webinars organized by researchers involved in the CBPR during the pandemic.

The last parameter considered was triple-negative BC. Data showed that triple-negative women, aware of their highly precarious health status, held a better PA level. Supplementary Tables 4–5 show the association between the abovementioned factors with MD score and WCRF adherence. In general, ORs estimates for MD and WCRF recommendations were not significantly different across strata.

## Discussion and conclusions

Since early 2020, to limit the spread of the COVID-19 pandemic, lockdown laws have imposed many different restrictions in several countries, thus influencing the lifestyle of the population, especially in terms of PA and diet. Many scientific organizations, such as WHO, have acknowledged the crucial role of PA and nutrition in both the prevention and treatment of nontransmissible chronic diseases. WHO published recommendations related to food and nutrition during the period of lockdown and some tips on how to stay active and reduce sedentary behavior [34, 35], since there is a close relationship between the quality of the diet and the overall health state, especially in patients with nontransmissible diseases, such as BC.

It is known that women who have received a BC diagnosis suffer throughout the entire process, including the diagnostic, therapeutic, and survival phases. In general, after surgical and/or clinical (antineoplastic chemotherapy, radiotherapy, endocrine therapy) intervention, post-treatment patients continue with consultations or follow-up visits for up to 10 years, regardless of endocrine therapy indication [36]. Some women may experience anxiety, depression, fatigue, pain, and sleep disturbance [37].

The COVID-19 pandemic may result in a higher worry for vulnerable BC survivors than their less vulnerable counterparts. During the pandemic, Seven et al. [38] reported that BC survivors expressed constant bone pain, increased fatigue, anxiety, feeling oversensitive, and weight gain.

In the DianaWeb study, tertiary prevention actions for BC survivors include healthy lifestyle strategies based on WCRF recommendation, as well as the achievement of a dietary pattern rich in vegetables, fruits, and whole grains (i.e., MD), the maintenance of a healthy body weight, and PA.

Diet may play a role in surviving a BC diagnosis: there are trends associating dietary fat with increased mortality [39, 40], whereas diets high in fibers were reported to have a weak benefit (HRs per 1 SD increment in intake: 0.98, 95% CI 0.95–1.00) [41]. Even if the association between diet or specific dietary components and breast cancer prognosis is weak and inconsistent, dietary polyphenols found in the MD have been reported to reduce inflammation and cancer recurrence through various mechanisms, including direct antioxidant activity or antioxidant gene expression, thus inhibiting cancer cell proliferation, cytokines and endotoxin-mediated kinases, and transcription factors involved in cancer progression or increasing histone deacetylase activity [42]. The MD might confer additional benefits to the BC survivors [28].

The strongest evidence for an effect on BC outcome was found for PA: women who participate in PA after a diagnosis of BC have reported a significant decrease in depression (Beck Depression Inventory, *p* < 0.01; Hamilton Depression Scale, *p* < 0.001) [43], and anxiety (ES –0.346; 95% CI –0.538 to –0.154, *p* < 0,001) [44], and improvements in body image (ES 0.280; 95% CI 0.077 to 0.482; *p* = 0.007) [44]. PA has been associated with better health outcomes for women diagnosed with BC, including reduced risk of BC recurrence, all-cause mortality, and BC mortality. Other benefits associated with PA for women diagnosed with BC include improvement in fatigue levels, physical functioning, functional quality of life, and quality of sleep [45].

Due to quarantine and containment measures adopted by the Italian government to control the spread of COVID-19, the practice of PA was subjected to significant restrictions (gyms were closed, and sport was canceled). Some surveys have highlighted that the COVID-19 pandemic has negatively affected PA levels [46–49]. To promote PA in the DianaWeb cohort, the effort comprised the implementation of the DianaWeb platform with specific coaching programs and specific webinars. Therefore, confinement did not cause substantial changes in total PA in the DianaWeb pandemic sub-cohort. As expected, we observed a significant decrease in walking activity during confinement compared with the period before. Further, during quarantine, we observed a decrease in the percentage of patients that declared to do vigorous PA. On the other hand, our results showed a significant increase in the percentage of women with a low PA level during lockdown (39.7% declared low levels of PA). During the quarantine, the women of the DianaWeb cohort were induced to modify their practices of PA in a home-based setting. However, in some cases, the women could be unable to adapt to their regular training at home. We suppose that among the possible causes for the observed lower levels of PA, the principal was a lack of equipment at home or the insufficiency of large spaces usually available for PA. In post quarantine total weekly energy expenditure (MET-min/week) increased significantly compared with the levels observed before COVID-19. It can be hypothesized that in post-lockdown women may have strengthened their autonomous motivation for PA (participating out of enjoyment, or the personal value attached), because of seeing the benefits and value of increasing their PA levels. Gradual easing of limitations (e.g., wearing a mask in open spaces was not mandatory during post-lockdown periods) and COVID-19 vaccination campaigns started in 2021 might have positively influenced the reported trends. Finally, regardless of the pandemic, people participating in the DianaWeb study for more than 3 years, who were older, with a higher education level, and with a triple-negative BC, were more likely to be more active.

In the DianaWeb cohort, the lockdown periods did not significantly lead to changes in meal patterns. We observed a small and not significant decrease in fresh vegetable intake. The reasons given for this decline in vegetable intake can possibly be explained by the reduced shopping frequency during the lockdown. It should be noted that, compared with data recorded at baseline (mean of MedDiet score 8.20 ± 1.88), we observed a significant increase in adherence to the MD in the DianaWeb cohort. Overall, data from this study showed that 2 months before the COVID-19 pandemic, the women enrolled in the DianaWeb study reached a high adherence to the MD (mean of MedDiet score before COVID-19 = 10.06 ± 2.29) and that it did not decrease during the confinement (mean of MedDiet = 9.75 ± 2.23; 9.92 ± 2.27).

In the present study, we also investigated if adherence to the WCRF lifestyle recommendations was affected by the COVID-19 pandemic. Comprehensive scores—such as the WCRF score—incorporating different indicators allow the evaluation of an overall healthy lifestyle. Our study did not reveal any significant difference in WCRF scores evaluated immediately before, during, and after lockdown periods in the DianaWeb pandemic sub-cohort. The comparison with data at baseline showed a significant increase in WCRF scores evaluated after quarantine. Several studies have shown that the maintenance of these positive changes is associated with the status of health-related quality of life among BC survivors [50, 51].

One of the most significant changes across society is the use of web-based technology. The online nature of the DianaWeb study allowed us to increase the specific health-promoting educational interventions in BC survivors during COVID-19 lockdowns. During the pandemic, our protocol provided weekly webinars to maintain a personal relationship with the BC patients. In our experience, an *ad personam* approach may have an important value as it allows a more incisive and direct relationship with patients and acts on personal or family dynamics that can be an obstacle to the continuation of the result.

This work has strengths and limitations. The main strength of this study is the availability of data regarding pre-quarantine lifestyle habits, which allowed us to assess changes in nutrition and lifestyle behaviors consequent to home isolation. A limitation of the study could lie in self-reporting of PA and dietary intake; however, previously published results suggest that self-reporting can be considered as satisfactorily accurate for patients in the DianaWeb study [52]. Another limitation of the study may be that some effects of the Italian lockdown might be revealed later than the short duration of this investigation was able to observe. Furthermore, a limitation is represented by the non-probabilistic nature of the sample, which precludes the generalizability of the results to the entire population of Italian BC patients. In addition, the analysis of lifestyle changes relied on self-reported questionnaires, which are subject to social desirability and memory bias.

In conclusion, the reported results show that, in subjects involved in this study, the proposed CBPR approach was useful in achieving durable lifestyle changes, that have not been undermined either during a perturbing event, such as the COVID-19 pandemic.

## Supplementary Information

Below is the link to the electronic supplementary material.

Supplementary file1 (PDF 197 KB)
