# Supplementary material for: How the COVID-19 pandemic has affected eating habits and physical activity in breast cancer survivors: the DianaWeb study
Source: J Cancer Surviv. 2022 Dec 13;17(4):974–85. doi: 10.1007/s11764-022-01294-w (PMC9745269; doi:10.1007/s11764-022-01294-w)
Supplement: Supplementary file 1 — Supplementary file1 (PDF 197 KB) [file 11764_2022_1294_MOESM1_ESM.pdf]

Supplementary Table 1 Breast cancer clinical characteristics of the full DianaWeb cohort and the DianaWeb CoVid-19 pandemic sub-cohort.

Breast cancer (BC) clinical characteristics	Full DianaWeb cohort [a]	DianaWeb pandemic sub-cohort	*p*
Years after BC diagnosis [b]	7.80 ± 4.82	7.36 ± 5.30	0.214 [d]
≤ 2 years [c]	191 (12.8)	28 (12.5)	1.000 [e]
3–4 years [c]	200 (13.4)	59 (26.3)	0.000 [e]
5–9 years [c]	614 (41.3)	68 (30.4)	0.020 [e]
≥ 10 years [c]	464 (31.2)	66 (29.5)	0.641 [e]
Missing data	18 (1.2)	3 (1.3)	
BC laterality			
Left breast	757 (50.9)	111 (49.6)	0.829 [e]
Right breast	678 (45.6)	104 (46.4)	0.772 [e]
Bilateral breast	44 (3.0)	6 (2.7)	1.000 [e]
Missing data	8 (0.5)	3 (1.3)	
T categories for BC			
T0	15 (1.0)	2 (0.9)	1.000 [e]
T1	69 (4.6)	8 (3.6)	0.604 [e]
T1a	58 (3.9)	10 (4.5)	0.715 [e]
T1b	186 (12.5)	32 (14.3)	0.519 [e]
T1c	504 (33.9)	96 (42.9)	0.014 [e]
T1mic	19 (1.3)	2 (0.9)	1.000 [e]
T2	329 (22.1)	35 (15.6)	0.017 [e]
T3	31 (2.1)	8 (3.6)	0.227 [e]
T4	5 (0.3)	0 (0.0)	1.000 [e]
T4b	5 (0.3)	0 (0.0)	1.000 [e]
T4d	1 (0.1)	0 (0.0)	1.000 [e]
Tis	78 (5.2)	9 (4.0)	0.515 [e]
TX	28 (1.9)	2 (0.9	0.416 [e]
Missing	159 (10.7)	20 (8.9)	
N categories for BC			
N0	726 (48.8)	119 (53.1)	0.450 [e]
N0(i-)	8 (0.5)	1 (0.4)	1.000 [e]
N0(i+)	1 (0.1)	0 (0.0)	1.000 [e]
N1	157 (10.6)	22 (9.8)	0.727 [e]
N1a	148 (10.0)	27 (12.1)	0.410 [e]
N1b	7 (0.5)	0 (0.0)	0.603 [e]
N1c	2 (0.1)	1 (0.4)	0.351 [e]
N1mic	75 (5.0)	7 (3.1)	0.242 [e]
N2	34 (2.3)	5 (2.2)	1.000 [e]
N2a	42 (2.8)	10 (4.5)	0.214 [e]
N3	16 (1.1)	1 (0.4)	0.717 [e]
N3a	29 (2.0)	2 (0.9)	0.420 [e]
N3b	1 (0.1)	0 (0.0)	1.000 [e]
NX	75 (5.0)	10 (4.5)	0.745 [e]
Missing	166 (11.2)	19 (8.5)	

Supplementary Table 1 (*Continued*)

Breast cancer (BC) clinical characteristics	Full DianaWeb cohort [a]	DianaWeb pandemic sub-cohort	*p*
M categories for BC			
M0	465 (31.3)	52 (23.2)	0.007 [e]
M1	29 (2.0)	4 (1.8)	1.000 [e]
MX	732 (49.2)	132 (58.9)	0.006 [e]
Missing	261 (17.6	36 (16.1	
Stage groups for breast cancer			
0	48 (3.2)	2 (0.9)	0.036 [e]
IA	446 (30.0)	90 (40.2)	0.008 [e]
IB	43 (2.9)	5 (2.2)	0.669 [e]
IIA	279 (18.8)	44 (19.6)	1.000 [e]
IIB	124 (8.3)	8 (3.6)	0.005 [e]
IIIA	66 (4.4)	16 (7.1)	0.134 [e]
IIIB	8 (0.5)	0 (0.0)	0.608 [e]
IIIC	39 (2.6)	3 (1.3)	0.259 [e]
IV	26 (1.7)	4 (1.8)	1.000 [e]
Missing	408 (27.4)	52 (23.2)	
Tumor grade [c]			
G1	152 (10.2)	24 (10.7)	1.000 [e]
G2	605 (40.7)	109 (48.7)	1.145 [e]
G3	520 (35.0)	66 (29.5)	0.024 [e]
G4	4 (0.3)	0 (0.0)	1.000 [e]
Gx	94 (6.3)	22 (9.8)	1.123 [e]
Missing data	112 (7.5)	3 (1.3)	
ER expression [c]			
Positive	1179 (79.3)	175 (78.1)	0.836 [e]
Missing data	90 (6.1)	19 (8.5)	
HER2 overexpression/amplification [c]			
Positive	506 (34.0)	90 (40.2)	0.281 [e]
Missing data	244 (16.4)	23 (10.3)	
PR expression [c]			
Positive	1042 (70.1)	157 (77.1)	0.786 [e]
Missing data	108 (7.3)	20 (8.9)	
Triple-negative breast cancer [c]			
Yes	353 (23.7)	63 (28.1)	0.338 [e]
Missing data	279 (18.8)	32 (14.3)	

[a] Subjects of the DianaWeb pandemic sub-cohort not included.
[b] Results expressed as the mean ± SD.
[c] Results expressed as the number of subjects, percentage between brackets.
[d] Student's *t* test: full DianaWeb cohort *vs* DianaWeb pandemic sub-cohort.
[e] χ^2 test: full DianaWeb cohort *vs* DianaWeb pandemic sub-cohort.

Supplementary Table 2 Sleep habits and smoking status at baseline and immediately before, during, and after lockdown periods in the DianaWeb pandemic sub-cohort.

	Baseline	Before CoViD-19 pandemic	During lockdown periods	After lockdown periods
Difficulty falling asleep [a]	50 (22.3)	37 (16.5)	47 (21.0)	40 (17.9)
Repeated awakenings [a]	116 (51.8)	102 (45.5)	92 (41.1)	110 (49.1)
Smoking status [b]				
Current	13 (5.8)	11 (4.9)	11 (4.9)	12 (5.4)
Cigarettes/day	20.15 ± 14.69	28.27 ± 18.28	23.36 ± 16.19	27.25 ± 19.10

[a] Results expressed as the number of subjects, percentage between brackets.

Supplementary Table 3 Factors associated with Physical Activity (PA) at baseline and during the Italian CoViD-19 pandemic in the DianaWeb sub-cohort.

	ß-coefficients (95% CI)			
	Baseline	Before CoViD-19 pandemic	During lockdown periods	After lockdown periods
Years from enrolment in the DianaWeb study				
≤ 2 years [a]	- - -	1	1	1
3-4 years	- - -	0.994 (0.986-1.002)	0.824 (0.816-0.831)	1.098 (1.090-1.106)
≥5 years	- - -	1.318 (1.305-1.330)	1.410 (1.397-1.424)	1.472 (1.460-1.484)
Area of residence				
Northern Italy [a]	1	1	1	1
Central Italy	1.183 (1.169-1.197)	1.114 (1.102-1.126)	1.293 (1.276-1.310)	1.091 (1.081-1.102)
Southern Italy	0.937 (0.923-0.952)	1.140 (1.126-1.153)	1.340 (1.326-1.354)	1.031 (1.019-1.043)
Age				
Young adults (aged 21–40) [a]	1	1	1	1
Adults (aged 41–60)	1.583 (1.546-1.621)	1.588 (1.555-1.621)	1.037 (1.018-1.057)	1.592 (1.560-1.623)
Over 60 aged	1.679 (1.638-1.722)	1.120 (1.095-1.146)	0.760 (0.744-0.775)	1.480 (1.450-1.511)
Marital status				
Never married [a]	1	1	1	1
Widowed	1.413 (1.379-1.447)	0.885 (0.859-0.912)	1.441 (1.402-1.480)	1.462 (1.428-1.496)
Separated/divorced	0.892 (0.876-0.909)	1.170 (1.152-1.188)	1.868 (1.841-1.896)	1.613 (1.592-1.634)
Married	1.120 (1.109-1.131)	1.049 (1.041-1.058)	1.102 (1.092-1.113)	1.343 (1.332-1.353)
Education level				
First level [a]	1	1	1	1
Second level	0.608 (0.599-0.617)	0.765 (0.753-0.778)	1.206 (1.181-1.231)	1.016 (1.001-1.032)
Higher education	0.684 (0.674-0.695)	1.097 (1.079-1.116)	1.347 (1.319-1.376)	1.516 (1.493-1.539)
Triple-negative breast cancer				
No [a]	1	1	1	1
Yes	1.078 (1.059-1.098)	1.456 (1.435-1.477)	1.147 (1.128-1.166)	1.881 (1.860-1.902)

[a] Reference category.

Supplementary Table 4 Factors associated with MD score at baseline and during the Italian CoViD-19 pandemic in the DianaWeb sub-cohort.

	ß-coefficients (95% CI)			
	Baseline	Before CoViD-19 pandemic	During lockdown periods	After lockdown periods
Years from enrolment in the DianaWeb study				
≤ 2 years [a]	- - -	1	1	1
3-4 years	- - -	0.987 (0.896-1.088)	0.993 (0.900-1.095)	0.961 (0.872-1.059)
≥5 years	- - -	1.002 (0.890-1.128)	0.979 (0.867-1.105)	0.978 (0.868-1.101)
Area of residence				
Northern Italy [a]	1	1	1	1
Central Italy	1.062 (0.923-1.222)	0.986 (0.866-1.123)	1.051 (0.924-1.197)	1.011 (0.888-1.152)
Southern Italy	1.050 (0.885-1.246)	0.978 (0.835-1.146)	0.992 (0.846-1.164)	0.986 (0.842-1.156)
Age				
Young adults (aged 21–40) [a]	1	1	1	1
Adults (aged 41–60)	0.881 (0.702-1.106)	0.990 (0.784-1.250)	0.932 (0.753-1.152)	0.916 (0.742-1.132)
Over 60 aged	0.936 (0.733-1.196)	0.977 (0.787-1.212)	0.958 (0.761-1.206)	0.943 (0.750-1.186)
Marital status				
Never married [a]	1	1	1	1
Widowed	0.939 (0.671-1.314)	0.917 (0.672-1.250)	0.935 (0.687-1.273)	0.905 (0.663-1.236)
Separated/divorced	1.018 (0.830-1.249)	1.062 (0.884-1.276)	0.933 (0.770-1.130)	0.977 (0.809-1.181)
Married	0.955 (0.853-1.070)	0.953 (0.859-1.056)	0.932 (0.840-1.034)	0.968 (0.872-1.073)
Education level				
First level [a]	1	1	1	1
Second level	0.984 (0.797-1.216)	0.978 (0.807-1.186)	1.012 (0.830-1.234)	0.929 (0.768-1.124)
Higher education	1.014 (0.816-1.259)	1.038 (0.852-1.265)	1.048 (0.855-1.284)	0.982 (0.807-1.194)
Triple-negative breast cancer				
No [a]	1	1	1	1
Yes	1.102 (0.885-1.371)	0.095 (0.900-1.333)	1.088 (0.890-1.330)	1.171 (0.965-1.422)

[a] Reference category.

Supplementary Table 5 Factors associated with WCRF score at baseline and during the Italian CoViD-19 pandemic in the DianaWeb sub-cohort.

	ß-coefficients (95% CI)			
	Baseline	Before CoViD-19 pandemic	During lockdown periods	After lockdown periods
Years from enrolment in the DianaWeb study				
≤ 2 years [a]	- - -	1	1	1
3-4 years	- - -	0.952 (0.823-1.101)	1.001 (0.865-1.157)	1.024 (0.887-1.183)
≥5 years	- - -	0.986 (0.825-1.178)	1.029 (0.862-1.229)	1.024 (0.859-1.222)
Area of residence				
Northern Italy [a]	1	1	1	1
Central Italy	1.019 (0.837-1.241)	1.039 (0.856-1.261)	1.026 (0.846-1.245	1.039 (0.858-1.259)
Southern Italy	1.059 (0.838-1.339	1.068 (0.848-1.346)	1.046 (0.831-1.317)	1.078 (0.859-1.353)
Age				
Young adults (aged 21–40) [a]	1	1	1	1
Adults (aged 41–60)	0.999 (0.717-1.390	1.044 (0.750-1.454)	1.032 (0.744-1.431)	1.043 (0.752-1.446)
Over 60 aged	0.988 (0.692-1.410	0.960 (0.671-1.374)	0.990 (0.695-1.410)	1.062 (0.747-1.510)
Marital status				
Never married [a]	1	1	1	1
Widowed	0.999 (0.628-1.589)	0.944 (0.593-1.504	0.976 (0.607-1.569)	0.996 (0.631-1.570)
Separated/divorced	1.053 (0.792-1.400)	1.045 (0.791-1.380	1.120 (0.850-1.478)	1.077 (0.819-1.418)
Married	0.992 (0.847-1.162)	0.956 (0.819-1.115	1.000 (0.856-1.168)	0.986 (0.846-1.150)
Education level				
First level [a]	1	1	1	1
Second level	1.001 (0.744-1.347)	0.977 (0.726-1.315	1.050 (0.777-1.419)	0.998 (0.747-1.332)
Higher education	1.032 (0.762-1.398)	1.003 (0.740-1.359	1.133 (0.832-1.541)	1.073 (0.798-1.444)
Triple-negative breast cancer				
No [a]	1	1	1	1
Yes	1.055 (0.776-1.435)	1.179 (0.884-1.572)	1.003 (0.738-1.364)	1.106 (0.825-1.483)

[a] Reference category.
